# Haters Gonna Hate, Trolls Gonna Troll: The Personality Profile of a Facebook Troll

**DOI:** 10.3390/ijerph18115722

**Published:** 2021-05-26

**Authors:** Haukur Freyr Gylfason, Anita Hrund Sveinsdottir, Vaka Vésteinsdóttir, Rannveig Sigurvinsdottir

**Affiliations:** 1 Department of Business, Reykjavik University, 102 Reykjavik, Iceland; 2 Department of Psychology, Reykjavik University, 102 Reykjavik, Iceland; a.sveins@gmail.com (A.H.S.); rannveigssig@gmail.com (R.S.); 3 Department of Psychology, University of Iceland, 102 Reykjavik, Iceland; vakav@hi.is

**Keywords:** trolling, personality, sadism, Dark Triad, HEXACO, antisocial internet behavior

## Abstract

Personality factors, such as the Dark Tetrad personality factors (Machiavellianism, narcissism and sadism) relate to greater online trolling. Other personality factors, such as the Big Five Personality factors, honesty–humility and negative social potency, may also play a role in cyberbullying, which is an aggressive behavior similar to trolling. The purpose of this study was to predict Facebook trolling behavior based on personality factors. A total of 139 participants completed a survey on their online behavior and personality factors. Online trolling behavior positively correlated with sadism, psychopathy and Machiavellianism, and negatively correlated with agreeableness, conscientiousness and honesty–humility. A hierarchical linear regression showed that sadism, Machiavellianism and negative social potency were the only unique predictors of online trolling behavior. Trolling was unrelated to the frequency of Facebook use and the frequency of commenting. Enjoyment of trolling fully mediated the relationship between Machiavellianism and the trolling behavior. The results thus suggested that Facebook trolling behaviors may be motivated by enjoying the manipulation of others.

## 1. Introduction

Trolling can be defined as a deviant, malicious or antisocial online behavior [1,2] with motives to disrupt conversations and trigger conflict [1,2,3]. The key aspects of trolling are deception, aggression, disruptive online activities and a sense of accomplishment when trolls get attention from others. Trolling shares similarities with cyberbullying [4] because both involve aggressive online behavior, but their targets vary, as trolls aim to create disruption among strangers, but cyberbullies target victims they know personally [5]. Internet trolling is a surprisingly common behavior, as up to a quarter of Americans admit having committed malicious online activity against a stranger [6], 33% in Malaysia [7], 11% in Hungary [8], and 3% had experienced some sort of online bullying, as reported in a review of victim surveys in Europe (Sweden, UK, The Netherlands, Germany, France and Luxembourg) [9]. Preventing and stopping trolling is particularly important because its victims face a negative psychological impact which is comparable to that of cyberbullying and in-person harassment [10,11,12].

### 1.1. Personality Traits and Trolling

Trolling has been studied separately within two personality frameworks: The Dark Tetrad and the Big Five [13]. The Dark Tetrad is a group of personality traits characterized by manipulating others and callousness [14]. For example, narcissism involves feeling that one is better than other people, and Machiavellianism is characterized by trying to control and manipulate others [15]. Psychopathy, on the other hand, includes impulsive behavior and a lack of empathy, whereas the fourth trait, sadism, is characterized by enjoying the suffering of others [16]. The Dark Tetrad personality traits are connected with a range of antisocial behaviors, such as bullying [17] and cyberbullying [18]. Additionally, the Dark Tetrad is connected with greater internet trolling, especially sadism [16,19] and psychopathy [19,20]. 

The Big Five personality framework includes extraversion, agreeableness, conscientiousness, neuroticism and openness [21]. These traits relate to antisocial behavior, as lower conscientiousness, lower agreeableness and greater extraversion are connected with trolling [13] and cyberbullying [22]. The Dark Tetrad and Big Five frameworks have already been integrated to study cyberbullying, which is connected with sadism and agreeableness [23,24]. However, no work to date has examined trolling in this context. Given the somewhat overlapping characteristics of cyberbullying and trolling, we believe that both personality frameworks could be relevant to trolling behavior.

In addition to the Dark Tetrad and the Big Five personality traits, another factor from the HEXACO Personality Inventory, honesty–humility, might be justified [25]. This is particularly relevant for antisocial behavior because the negative pole of the honesty–humility factor could indicate a tendency for exploiting people [26]. Furthermore, honesty–humility predicts dishonest, deceitful and antisocial behavior [27,28]. This trait is unique because it may involve personally exploiting others, but only for direct, personal gain. The Dark Tetrad, on the other hand, characterizes individuals who enjoy the suffering of others [16]. Currently, work is lacking on the intersection between these personality frameworks and trolling.

### 1.2. The Social Aspect of Trolling

In addition to personality traits, social factors also explain trolling behavior, as trolls crave recognition from others to feel successful [5]. Social rewards are factors in the social environment that reinforce different types of behavior, either for being socially accepted (typical social rewards) or not (atypical social rewards). Negative social potency is a form of an atypical social reward, usually connected with selfish behavior [29]. People who pursue negative social potency are likely to be cruel, callous and abusing [30]. Negative social potency is predictive of online trolling behavior [19] and those who troll others may therefore be motivated to harm others by wielding negative social influence. 

### 1.3. Current Study

The purpose of this study was to predict Facebook trolling behavior based on personality factors. Previous studies have shown that the Dark Tetrad personality traits help explain trolling [13,19] and that the Big Five personality traits may also contribute to trolling [13]. However, we intended to expand the current literature by integrating these two frameworks along with assessment of the role that honesty–humility plays in trolling. Furthermore, we intended to examine the role that negative social potency plays in trolling, in accordance with previous literature [19]. Frequency of internet use has also been hypothesized as an important factor in trolling because heavy users have greater opportunity to troll [13] or cyberbully [18,31] and because heavy use has been connected with high extraversion and low agreeableness and conscientiousness [32]. Because of this, we intended to control for frequency of Facebook use in our analyses. Additionally, we controlled for a key social dimension, impression management, given that a socially desirable response style can have confounding effects on self-reported data, leading to distorted information [33,34,35]. We hypothesized that trolling behavior would be positively associated with higher levels of Dark Tetrad personality traits [13,19], positively associated with negative social potency [19], positively associated with extraversion, negatively associated with agreeableness and conscientiousness [13], and negatively associated with honesty–humility [26]. Furthermore, we hypothesized that, in addition to the variance explained by the Dark Tetrad traits and negative social potency, HEXACO personality traits would also predict Facebook trolling behavior. We further expected that the Dark Tetrad would be positively associated with the enjoyment of trolling. 

## 2. Material and Methods

### 2.1. Participants and Procedure

To determine the required sample size, an a priori power analysis was conducted. Based on previous research [19], a probable effect size when assessing the association between Facebook trolling behavior and the Dark Tetrad traits was thought to be medium sized. To detect a similar effect (Pearson’s *r* = 0.35, with acceptable statistical power (1 − *β*) = 0.80), a sample size of *N* = 46 (one-sided) was required [36,37]. 

A total of 147 participants participated in an online survey in May 2018. Eight participants failed to respond correctly to three data quality monitoring questions and were, therefore, removed from the data. Out of the 139 participants left, 17 (12.2%) were male, 119 (85.6%) were female and 3 (2.2%) were non-binary. Participant age varied, as 35 participants (25.2%) were under 25 years old, 72 (51.8%) were 25 to 34 years of age, 17 (12.2%) were 35 to 44 years of age, 6 (4.3%) were 45 to 54 years of age, and 9 (6.4%) were older than 54 years old. 

Participants were recruited through Facebook using a snowball sample where participants were asked to share the survey with their friends on Facebook. The inclusion criteria for the study were being at least 18 years old and to have a Facebook account. Informed consent was obtained from each subject. Participants were informed of their rights as research participants, that their participation was voluntary, their responses strictly confidential, that they did not need to answer all questions, and could leave the survey at any time if they so wished. No reward was offered for participation. The APA ethical principles and code of conduct guided our research and we followed the principles of the Declaration of Helsinki.

### 2.2. Measures

The survey included demographic questions (age and gender). In addition, the following measures were used: 

The Global Assessment of Facebook Trolling. To assess participants’ Facebook trolling behavior, the Global Assessment of Facebook Trolling (GAFT) was used [19]. Participants rated how much they agreed with statements such as “I like to disrupt and derail people in comment sections or newsfeeds on Facebook” on a five point Likert scale ranging from 1 (strongly disagree) to 5 (strongly agree). The scale was found to have reasonable reliability in the current sample (Cronbach’s α = 0.67). 

The Dirty Dozen. Machiavellianism, narcissism and psychopathy were measured with The Dirty Dozen, a 12 item self-report questionnaire [38]. Statements such as “I tend to be callous or insensitive” and “I tend to seek prestige or status” were rated on a 9 point Likert scale from 1 (disagree strongly) to 9 (agree strongly). Previous work has supported the reliability and validity of the measure [38]. In the current sample, internal consistency was good for the overall measure (Cronbach’s α = 0.87) and subscales: Machiavellianism α = 0.80, narcissism α = 0.86, and psychopathy α = 0.74. 

The Short Sadistic Impulse Scale. The Short Sadistic Impulse Scale (SSIS) was used to measure sadistic inclination [39]. Participants rated 10 statements such as “I have hurt people because I could” on a dichotomous scale as “Unlike me” or “Like me”. In the current sample, the SSIS was found to have fairly low reliability (Cronbach’s α = 0.63), similar to that in Craker and March [19]. 

Social Rewards Questionnaire. Participants’ negative social potency was measured using the Social Rewards Questionnaire (SRQ) [30]. Participants rated 23 statements on a 7 point Likert scale ranging from 1 (Disagree strongly) to 7 (Agree strongly). Examples of statements are “I enjoy making someone angry” and “I enjoy embarrassing others”. The questionnaire measures six subscales of social reward: admiration, negative social potency, passivity, prosocial interactions, sexual reward and sociability. Negative social potency was the only subscale of interest for the current study. The Social Rewards Questionnaire had been shown to have good reliability and construct validity [30]. Reliability for the current sample was found to be good for the SRQ, α = 0.79, and for the subscale negative social potency, α = 0.64. 

HEXACO Personality Inventory Revised. The HEXACO Personality Inventory Revised (HEXACO-PI-R) was used to assess honesty–humility, emotionality, extraversion, agreeableness, conscientiousness and openness [25]. Statements such as “I would be quite bored by a visit to an art gallery” and “I would get a lot of pleasure from owning expensive luxury goods” were answered on a 5 point Likert scale from 1 (strongly disagree) to 5 (strongly agree). Previous work has supported the reliability and validity of this measure [25]. Reliability of the subscales was found to be good (honesty–humility: α = 0.70, emotionality: α = 0.75, extraversion: α = 0.87, agreeableness: α = 0.74, conscientiousness: α = 0.76 and openness: α = 0.80). 

Impression management. The short form of the impression management subscale of the Balanced Inventory of Desirable Responding was used to assess participants “deliberate self-presentation” [40] (p. 37). The validity and reliability of this measure had been supported by previous research [33]. Participants rated 12 statements, such as “I sometimes tell lies if I have to“, with a partly labelled seven point answer scale from 1 (not true), 4 (somewhat true) to 7 (very true). The reliability of the scale was good, α = 0.74. 

Facebook activities. In the section on Facebook use, we asked participants “How many hours per day do you spend on Facebook?”, and if participants posted comments on Facebook, and if so, for how many hours per day did they spend posting comments on Facebook. Additionally, participants rated their enjoyment of the following activities on Facebook—debating, chatting, trolling, and making friends—on a five-point scale from 1 (not at all enjoyable) to 5 (very enjoyable) [13]. 

### 2.3. Data Analysis

SPSS, version 26 (IBM, Armonk, NY, USA), and Mplus, version 6.12 (Muthen & Muthen, Los Angeles, CA, USA), were used to perform the analyses. We performed a descriptive statistical analysis for all personality variables using mean, standard deviation, and Cronbach’s alpha. To examine the relationships between trolling behavior and the personality measures, Pearson correlation and multiple linear regression were used. In all analyses, we ascertained that the assumptions were met. The minimum alpha level for significance was set at α = 0.05. The path analysis was performed with 5000 bootstrapped samples and the fit of the path model was good (CFI = 0.98, SRMR = 0.04) [41]. 

## 3. Results

Mean scores, standard deviations, reliability coefficients and correlation coefficients for the personality variables and Facebook trolling behaviors are reported in Table 1. As predicted, trolling behavior was positively associated with higher levels of the Dark Tetrad personality traits, except for narcissism (*p* > 0.05). Trolling was also negatively associated with honesty–humility, agreeableness and conscientiousness as predicted, but also negatively associated with extraversion. Negative social potency had a positive relationship with trolling. There was no gender difference in participants’ Facebook trolling behavior, *t*(145) = 0.90, *p* = 0.37. 

To test whether the HEXACO personality traits predicted Facebook trolling behavior, in addition to the Dark Tetrad personality traits and negative social potency, we ran a hierarchical linear regression analysis (see Table 2). The independent variables were entered simultaneously after it was ascertained that no assumptions were violated, including the assumption of multicollinearity. In the first step (Model 1), gender, age, time spent on Facebook, and impression management were entered as independent variables. In the second step (Model 2), the Dark Tetrad personality traits were added as independent variables. In the third step (Model 3), negative social potency was added as an independent variable, and in the fourth step (Model 4), the HEXACO personality traits were added as independent variables. In Model 1, only impression management was a negative predictor of Facebook trolling behavior. In Model 2, sadism and Machiavellianism were both positive predictors of Facebook trolling behavior and in Model 3 sadism, Machiavellianism and negative social potency were positive predictors of Facebook trolling. Finally, with the addition of the HEXACO personality traits in Model 4, sadism, Machiavellianism, negative social potency and conscientiousness were significant predictors of Facebook trolling behavior. 

At each step in the regression, apart from the last one, there was a significant improvement in the model fit. This implies that the Dark Tetrad and negative social potency improved the prediction of Facebook trolling behavior. The HEXACO personality variables did not, with the exception of conscientiousness, significantly predict Facebook trolling behavior when controlling for previously added variables. For these variables, multicollinearity was not a concern, as tolerance scores were higher than 0.4, VIF scores were below 2.2 and correlations between variables were not strong enough to cause issues with multicollinearity (above 0.80) [42,43]. According to Cohen [44], the effect size of Model 1 (*ƒ*^2^ = 0.10) was small, and large for Model 2 (*ƒ*^2^ = 0.38), Model 3 (*ƒ*^2^ = 0.43) and Model 4 (*ƒ*^2^ = 0.52). 

Correlation coefficients for Facebook trolling behaviors, the Dark Tetrad and enjoyment of Facebook activities are displayed in Table 3. Predictably, Facebook trolling behaviors were positively associated with trolling enjoyment. As expected, the Dark Tetrad scores were positively associated with commenting frequency on Facebook, but commenting frequency was not connected to trolling behavior. Self-reported enjoyment of trolling was positively associated with sadism and Machiavellianism. Enjoyment of debating on Facebook was associated with psychopathy, sadism and Machiavellianism. Finally, enjoyment of Facebook chatting was connected to narcissism. 

Given that self-reported enjoyment of trolling was connected with both trolling and personality factors, we tested whether enjoyment acted as a mediator between personality variables and trolling behavior. Using structural equation modeling, we constructed a path model with the factors previously identified as most important for trolling (Machiavellianism, sadism and conscientiousness, in addition to negative social potency) as exogenous variables, trolling enjoyment as a mediator and trolling behavior as the endogenous variable. We found that the relationship previously identified between Machiavellianism and trolling behavior was fully mediated through trolling enjoyment (standardized coefficient for indirect effect = 0.08, *p* = 0.04). The other factors were connected with trolling behavior (sadism; *β* = 0.26, *p* < 0.001, negative social potency; *β* = 0.19, *p* < 0.001, conscientiousness: *β* = −0.18, *p* < 0.001), but not with the enjoyment of trolling. 

## 4. Discussion

This study examined the association between the Dark Tetrad personality factors, the HEXACO traits, negative social potency and Facebook trolling behaviors. In addition, the aim was to study whether the HEXACO traits predicted trolling when the Dark Tetrad and negative social potency were controlled for. Finally, we examined the relationship between these personality and social factors, not just with Facebook trolling behavior, but also with enjoyment of various online activities. 

In line with our hypotheses, Machiavellianism, psychopathy and sadism positively correlated with Facebook trolling behavior, where sadism was the strongest predictor. This is consistent with previous studies on trolling [13,19] and cyberbullying [18,24]. Narcissism had a positive, although non-significant, association with trolling, which is in line with other studies [19,24]. As in previous work, Facebook trolling behavior was negatively associated with agreeableness and conscientiousness [13,24], positively associated with negative social potency [19] and negatively associated with honesty–humility. 

Unexpectedly, the relationship between Facebook trolling behaviors and extraversion was negative. Other scholars have speculated whether trolling and extraversion may have a positive relationship [13,45,46], due, in most part, to the positive association between extraversion and high internet use [47] and greater online antisocial behavior among men [19]. However, other studies have not found a link between extraversion and cyberbullying, indicating that a negative association could also be supported [24]. These findings suggest that because Facebook is an online platform where anonymity is more difficult to sustain, trolling on that site is less an impulsive behavior and more a calculative, planned one. The findings regarding sadism and Machiavellianism also reinforce this point. 

Sadism, Machiavellianism and negative social potency relate to greater trolling behavior and conscientiousness to less trolling behavior, when controlling for other personality factors. This is in line with previous studies, where sadism was a unique predictor of trolling behaviors [13,19] and cyberbullying [24]. A similar pattern has emerged for negative social potency [19]. However, the picture is less clear for conscientiousness and Machiavellianism. This is the first study where conscientiousness was found to predict Facebook trolling behaviors when controlling for the Dark Tetrad and the HEXACO traits. However, it should be noted that sadism had the strongest relationship with trolling in our study. Traditional bullying has a negative association with conscientiousness [46,48], but conscientiousness has not previously predicted cyberbullying [24]. Conscientiousness could be related to trolling behaviors through empathy, given that conscientiousness is one of the personality dimensions most associated with empathy [49] and online trolling behaviors are associated with low empathy [50]. This needs to be studied further. Additionally, in this study, Machiavellianism was a unique predictor of trolling behavior, contrary to previous works on this topic [18,19,24]. However, given that manipulation is a key ingredient of Machiavellianism, this is hardly surprising. Even though honesty–humility was related to trolling behaviors at the bivariate level, it became non-significant in the regression analysis, which suggested that this personality factor might play a smaller role in such an undesirable behavior as trolling, as its previous conceptualization would suggest.

In order to better understand the motivation of those who troll, we asked participants what kinds of activities on Facebook they enjoyed taking part in. Commenting frequency was not connected with trolling behavior, contrary to previous work [13], suggesting that for our participants, trolling was a planned behavior rather than determined by having an opportunity to troll. Enjoying trolling was positively connected with sadism and Machiavellianism [13] and sadism, psychopathy and Machiavellianism were also connected with the enjoyment of debating. We also found that narcissism was positively associated with the enjoyment of chatting. This differs slightly from previous work, which has found psychopathy to be positively related to chatting and narcissism to be connected with debating [13]. Our mediation analysis showed that the relationship between Machiavellianism and trolling behavior was fully mediated by the enjoyment of trolling. Therefore, it is possible that those high in this trait troll simply because they like to do so. The motivation to troll for Machiavellian purposes (to control and manipulate others) can therefore be understood in the context of simply taking pleasure out of the activity. For the other variables connected with trolling, motivation may differ. 

In these analyses, there were no differences by gender, which is inconsistent with previous research on trolling [19] and cyberbullying [24]. Among children, the effect of gender on cyberbullying is unclear, with some studies reporting gender differences [51,52] but not others [53]. In adult samples, perpetrators of cyberbullying are more commonly male [24,54]. For the present study, however, the lack of a gender difference could be an artefact of this Icelandic sample, which was mostly female, or it could be cultural because there appear to be no gender differences in deception in Iceland [55], as deception is a characteristic of trolling behaviors [5]. 

Some limitations of the current study include the use of a small sample with an uneven gender distribution, which may have impacted the results [19,54]. The sample only consisted of Icelandic speakers, a very small community, which may have impacted the extent to which people troll others and whether they are willing to disclose that behavior. Furthermore, all variables were based on self-report. Although we controlled for impression management, a socially desirable response style can have confounding effects on self-reported data, leading to distorted information [33,34,35]. In addition, reliability of certain scales could have been better in the current study. 

Future research should focus on exploring some of the mechanisms leading from these personality factors to bullying behaviors, such as through empathy. Attempting to boost protective individual factors could act as trolling prevention. In this context, a further examination of the HEXACO subscales in relation to trolling could prove fruitful. Alternatively, engaging in other forms of prevention and intervention could also be effective. For example, interventions where peers intervene in person to stop trolling behavior are promising [56], as well as online bystanders intervening [57]. For example, early studies show that a computer game targeted at online bystander skills is effective in increasing intention and self-efficacy to stop cyberbullying, but these effects need to be studied in the context of cyberbullying behavior [57]. Furthermore, future research should pay attention to the online platforms where antisocial behavior takes place, as their organization and different levels of anonymity may draw out different kinds of behavior. 

## 5. Conclusions

The results of the current study suggested that personality factors may play a role in explaining trolling behavior, but that personality also intersects with other important factors, such as social rewards and enjoyment of trolling. Interventions to decrease trolling could, therefore, target specific factors, such as decreasing troll enjoyment by blocking trolls from websites or discouraging other users from engaging with the trolls. Decreasing the power that trolls have only becomes more important with time, as online spaces represent an ever-growing part of the lives of the general public. This could also mean that as general users become more internet savvy, trolls could be stopped earlier than before, by being reported to moderators on the websites in question. 

## Figures and Tables

**Table 1:** Means, standard deviations, Cronbach’s alphas (α) and Pearson correlation coefficients for Facebook trolling behavior, impression management, Dark Tetrad traits, negative social potency and the HEXACO personality traits.

		M	SD	α	1	2	3	4	5	6	7	8	9	10	11	12
1.	Facebook trolling behaviors	18.30	50.53	0.66												
2.	Impression management	3.44	00.94	0.74	−0.279 **											
3.	Sadism	0.96	10.33	0.62	0.449 ***	−0.369 ***										
4.	Machiavellianism	3.21	10.65	0.78	0.367 ***	−0.547 ***	0.342 ***									
5.	Psychopathy	2.58	10.55	0.74	0.285 **	−0.305 ***	0.315 ***	0.551 ***								
6.	Narcissism	4.14	10.90	0.86	0.105	−0.301 ***	0.153	0.466 ***	0.377 ***							
7.	Negative social potency	1.73	00.75	0.63	0.417 ***	−0.371 ***	0.471 ***	0.403 ***	0.393 ***	0.287 **						
8.	Honesty−humility	3.43	00.59	0.69	−0.261 **	0.544 ***	−0.292 **	−0.471 ***	−0.390 ***	−0.515 ***	−0.443 ***					
9.	Extraversion	3.19	00.81	0.87	−0.188 *	0.169 *	−0.174 *	0.074	−0.010	0.203 *	−0.159	−0.056				
10.	Agreeableness	3.18	00.60	0.76	−0.312 ***	0.420 ***	−0.358 ***	−0.365 ***	−0.399 ***	−0.147	−0.326 ***	0.336 ***	0.202 *			
11.	Conscientiousness	3.51	00.62	0.77	−0.308 ***	0.291 **	−0.207 *	−0.265 **	−0.277 **	−0.172 *	−0.343 ***	0.319 ***	0.165	0.202 *		
12.	Openness	3.54	00.68	0.80	0.016	0.123	0.099	0.126	0.172 *	0.088	−0.134	0.065	0.215 *	0.100	−0.008	
13.	Emotionality	3.36	00.63	0.75	−0.029	−0.104	0.021	0.016	−0.077	0.079	0.121	−0.094	−0.261 **	−0.190 *	−0.083	−0.169 *

* *p* < 0.05, ** *p* < 0.01, *** *p* < 0.001.

**Table 2:** Summary of the hierarchical regression analysis predicting Facebook trolling behaviors.

	b	SE	β	Δ*R*^2^
Model 1				Δ*R*^2^ = 0.09, *F*(4, 134) = 3.24, *p* < 0.05
Constant	22.41 ***	2.42		
Male	1.15	1.41	0.068	
24 years of age or younger	0.83	1.06	0.066	
Hours per day on Facebook	0.21	0.33	0.053	
Impression management	−1.54 **	0.49	−0.262	
Model 2				Δ*R*^2^ = 0.19, *F*(4, 130) = 8.32, *p* < 0.001
Constant	14.38 ***	3.02		
Male	1.31	1.29	0.078	
24 years of age or younger	1.10	0.98	0.087	
Hours per day on Facebook	0.12	0.30	0.031	
Impression management	−0.08	0.54	−0.014	
Sadism	1.48 **	0.35	0.356	
Machiavellianism	0.81 *	0.36	0.242	
Psychopathy	0.22	0.33	0.063	
Narcissism	−0.32	0.25	−0.110	
Model 3				Δ*R*^2^ = 0.03, *F*(1, 129) = 4.91, *p* < 0.05
Constant	12.91 ***	3.05		
Male	0.96	1.28	0.057	
24 years of age or younger	0.92	0.97	0.073	
Hours per day on Facebook	0.01	0.30	0.002	
Impression management	0.03	0.54	0.005	
Sadism	1.21 **	0.37	0.290	
Machiavellianism	0.74 *	0.35	0.221	
Psychopathy	0.11	0.33	0.030	
Narcissism	−0.36	0.25	−0.123	
Negative social potency	1.49 *	0.67	0.204	
Model 4 ^a^				Δ*R*^2^ = 0.04, *F*(6, 123) = 1.22, *p* = 0.30
Constant	25.93 ***	6.74		
Male	0.66	1.32	0.039	
24 years of age or younger	0.62	1.00	0.049	
Hours per day on Facebook	−0.02	0.31	0.004	
Impression management	0.47	0.61	0.081	
Sadism	1.07 **	0.38	0.258	
Machiavellianism	0.79 *	0.36	0.235	
Psychopathy	−0.14	0.35	−0.038	
Narcissism	−0.24	0.27	−0.083	
Negative social potency	1.18 ^†^	0.72	0.161	
Honesty–humility	−0.28	0.98	−0.030	
Extraversion	−0.77	0.59	−0.113	
Agreeableness	−0.90	0.83	−0.098	
Conscientiousness	−1.27 ^†^	0.73	−0.144	
Openness	−0.01	0.68	0.002	
Emotionality	−0.85	0.73	−0.097	

^†^*p* ≤ 0.10, * *p* < 0.05, ** *p* < 0.01, *** *p* < 0.001. ^a^ Total explained variance in Model 4: Adjusted *R*^2^ = 0.26, *F* (15, 123) = 4.22, *p* < 0.001.

**Table 3:** Associations of Facebook trolling behaviors and Dark Tetrad variables with commenting behavior and enjoyment.

		1	2	3	4	5	6	7	8	9	10
1.	Facebook trolling behaviors										
2.	Sadism	0.449 ***									
3.	Machiavellianism	0.367 ***	0.342 ***								
4.	Psychopathy	0.285 **	0.315 ***	0.551 ***							
5.	Narcissism	0.105	0.153	0.466 ***	0.377 ***						
6.	Facebook use (h/day)	0.095	0.146	0.095	0.076	0.135					
7.	Commenting frequency (h/day)	0.042	0.175 *	0.208 *	0.186 *	0.179 *	0.233 **				
8.	Enjoyment of debating	0.294 ***	0.295 ***	0.356 ***	0.369 ***	00.161	0.022	0.347 ***			
9.	Enjoyment of chatting	−0.134	−0.077	0.062	0.001	0.233 **	0.237 **	0.121	−0.008		
10.	Enjoyment of trolling	0.551 ***	0.269 **	0.340 ***	0.052	0.045	0.041	−0.031	0.127	0.018	
11.	Enjoyment of making friends	0.052	0.107	0.080	−0.007	0.138	0.096	0.131	0.161	0.355 ***	0.084

* *p* < 0.05, ** *p* < 0.01, *** *p* < 0.001.

## Data Availability

The data presented in this study are available on request from the corresponding author. The data are not publicly available due to ethical restrictions.
